# Fetuin-A and Albumin Alter Cytotoxic Effects of Calcium Phosphate Nanoparticles on Human Vascular Smooth Muscle Cells

**DOI:** 10.1371/journal.pone.0097565

**Published:** 2014-05-21

**Authors:** Yana Dautova, Diana Kozlova, Jeremy N. Skepper, Matthias Epple, Martin D. Bootman, Diane Proudfoot

**Affiliations:** 1 Signalling ISP, Babraham Institute, Babraham, Cambridge, United Kingdom; 2 Inorganic Chemistry and Center for Nanointegration Duisburg-Essen (CeNIDE), University of Duisburg-Essen, Essen, Germany; 3 Cambridge Advanced Imaging Centre, Anatomy Building, University of Cambridge, Cambridge, United Kingdom; 4 Department of Life, Health and Chemical Sciences, The Open University, Milton Keynes, United Kingdom; University of California, Los Angeles, United States of America

## Abstract

Calcification is a detrimental process in vascular ageing and in diseases such as atherosclerosis and arthritis. In particular, small calcium phosphate (CaP) crystal deposits are associated with inflammation and atherosclerotic plaque de-stabilisation. We previously reported that CaP particles caused human vascular smooth muscle cell (VSMC) death and that serum reduced the toxic effects of the particles. Here, we found that the serum proteins fetuin-A and albumin (≥1 µM) reduced intracellular Ca^2+^ elevations and cell death in VSMCs in response to CaP particles. In addition, CaP particles functionalised with fetuin-A, but not albumin, were less toxic than naked CaP particles. Electron microscopic studies revealed that CaP particles were internalised in different ways; via macropinocytosis, membrane invagination or plasma membrane damage, which occurred within 10 minutes of exposure to particles. However, cell death did not occur until approximately 30 minutes, suggesting that plasma membrane repair and survival mechanisms were activated. In the presence of fetuin-A, CaP particle-induced damage was inhibited and CaP/plasma membrane interactions and particle uptake were delayed. Fetuin-A also reduced dissolution of CaP particles under acidic conditions, which may contribute to its cytoprotective effects after CaP particle exposure to VSMCs. These studies are particularly relevant to the calcification observed in blood vessels in patients with kidney disease, where circulating levels of fetuin-A and albumin are low, and in pathological situations where CaP crystal formation outweighs calcification-inhibitory mechanisms.

## Introduction

The biosynthesis of calcium phosphate (CaP) crystals is a tightly regulated physiological process occurring in bones and teeth. However, deposition of CaP crystals at non-skeletal locations (ectopic calcification) occurs in blood vessels in association with ageing and also in several diseases including atherosclerosis, arthritis and cancer. The amount of calcification in arteries correlates positively with the degree of atherosclerosis, and the presence of calcification in the intima or in the medial layer of blood vessels is detrimental [1]. Excessive calcification of the intima and/or media is a very common feature in diabetes and chronic kidney disease and predicts an increased risk for cardiovascular events and mortality. Calcification has been regarded as a silent marker of disease, but CaP crystals are known to be damaging in inflamed joints, and there is emerging evidence supporting a pro-inflammatory, destabilising effect of small CaP crystals in atherosclerosis [2]–[5]. Indeed, small CaP crystals (generally less than 5 µm) and other forms of particles have been shown to stimulate various cell signalling pathways resulting in profound effects on cell function, including proliferation, matrix degradation, cytokine secretion and cell death [6], [7]. CaP particles in arteries are found within or near vascular smooth muscle cells (VSMCs) and inflammatory cells. The micro- and nanocrystalline deposits are particularly associated with areas of atherosclerotic plaque stress and rupture, possibly because of engulfment by phagocytic cells causing inflammation and also via macrophage and VSMC death.

We have previously reported that CaP particles, either extracted from human calcified blood vessels or synthetic nanoparticles, induced VSMC death *in vitro* [8]. VSMC death in blood vessels is expected to have several consequences: to encourage inflammation via factors produced from apoptotic and necrotic cells; to weaken the fibrous cap enclosing atherosclerotic plaques due to loss of matrix-producing VSMCs; and to promote further calcification via generation of nucleation sites on dead cells. These effects could contribute to atherosclerotic plaque destabilisation and plaque rupture, leading to myocardial infarction or stroke.

Our previous studies found that CaP particles isolated from calcified atherosclerotic tissue were less potent in inducing cell death compared with synthetic CaP particles [8]. In addition, serum appeared to reduce the cytotoxic effects of synthetic CaP. We hypothesised that the observed reduced potency of the human-derived particles was due to their association with serum proteins such as fetuin-A and albumin [9], [10]. The fetuin family comprises two members: fetuin-A (originally termed ‘fetuin’ and also known as α2-Heremans Schmid glycoprotein) and fetuin-B that are highly expressed, circulating liver-derived proteins [11]. Both are members of the cystatin superfamily of protease inhibitors. Fetuin-A is a potent inhibitor of calcification [12] and fetuin-B has a critical role in fertilisation [13]. In mice lacking fetuin-A, extensive calcification occurs in soft tissues indicating that circulating fetuin-A is required to prevent calcification. In studies of patients with chronic kidney disease, low levels of circulating fetuin-A have been associated with increased artery calcification and higher mortality rates [14], [15]. Fetuin-A has a high affinity for hydroxyapatite crystals and is thought to function by binding small CaP particles via a domain particularly rich in acidic residues, stabilising and clearing them to phagocytes for removal [16]. Fetuin-A also has an anti-inflammatory function, dampening the effects of CaP particles in neutrophil stimulation, and also in macrophage cytokine release and induction of apoptosis [17], [18]. Additionally, fetuin-A has been shown to accumulate in VSMC-derived matrix vesicles, preventing them from initiating and propagating calcification [19]. The function of albumin in calcification is less clear in comparison with fetuin-A, but albumin has been described as a mineral chaperone, mediating the clearance of calciprotein particles (CPPs) [20]. We therefore aimed to determine whether fetuin-A or albumin could affect CaP particle-induced VSMC death.

## Materials and Methods

### Ethics Statement

Patients gave written informed consent for tissue samples to be used for research, on a standard hospital consent form. Ethical approval for use of human VSMC cultures was approved by the Cambridgeshire 1 Research Ethics Committee.

### Cell Culture

Human arterial VSMCs were grown and maintained in M199 (Sigma) containing 20% foetal bovine serum (PAA), buffered with 3.7 mg/mL NaHCO_3_ and 5% CO_2_ and supplemented with 100 IU/mL penicillin, 100 mg/mL streptomycin and 4 mM L-glutamine (Sigma). VSMCs were isolated from the medial layer of arterial tissue and were kindly provided by Prof. Martin Bennett (Addenbrooke’s Hospital, Cambridge). In some experiments, human VSMCs purchased from Lonza were cultured in SM-basal medium (Lonza) supplemented with 5% foetal bovine serum, insulin, human fibroblast growth factor, human epidermal growth factor and gentamycin. Cells were used between passages 3 and 15.

### Materials and Nanoparticles

Non-functionalised (CaP particles without attached chemicals or proteins) and protein-functionalised CaP nanoparticles (CaP particles with fetuin-A or albumin attached) were prepared by fast pumping of an aqueous solution of calcium lactate (9.0 mM; Merck) and an aqueous solution of diammonium hydrogen phosphate (5.4 mM; Merck). The pH of both solutions was adjusted to 8.0 by NaOH and afterwards sterile filtered through Filtropur S plus (0.2 µM). The precipitation reaction was achieved by rapidly pumping (5 mL/min) both solutions into a glass vessel under sterile conditions. Protein-functionalised CaP nanoparticles were prepared by simultaneous addition of 2 mL/min of aqueous protein solutions. The nanoparticles were functionalised with either fetuin-A from foetal bovine serum (1 mg/mL, suitable for cell culture, Sigma) or albumin from bovine serum, (1 mg/mL; suitable for cell culture, Serva). Then the prepared suspensions of nanoparticles with protein were immediately centrifuged at 900 rpm for 3 min for non-functionalised and albumin-functionalised nanoparticles and at 4000 rpm for 3 min for fetuin-A-functionalised nanoparticles. The supernatant was removed and the nanoparticles (CaP+protein) were resuspended in 200 times less water volume than the initial nanoparticle dispersion (see Table 1 for explanation of particle abbreviations).

**Table 1:** Explanation of particle abbreviations.

CaP	Non-functionalised or ‘naked’ calcium phosphate particles
CaP+fetuin-A	CaP particles in a solution containing fetuin-A
CaP+albumin	CaP particles in a solution containing albumin
CaP/F	CaP particles functionalised with fetuin-A
CaP/A	CaP particles functionalised with albumin

The concentration of Ca^2+^ in synthesised CaP nanoparticles was quantified by atomic absorption spectroscopy (AAS; M-Serie, Thermo Electron). The concentrations of particles used in this study are stated as mg/mL in terms of Ca^2+^ content, rather than weight of the nanoparticles. The content of calcium phosphate may be estimated by assuming the stoichiometry of hydroxyapatite, Ca_5_(PO_4_)_3_OH, and multiplying the calcium content by *M*(hydroxyapatite)/(5.*M*(Ca)) = 502/200 = 2.51. The morphology of freshly prepared nanoparticles was characterised with scanning electron microscopy (SEM; ESEM Quanta 400 FEG, gold/palladium sputtering). The particle diameter was between 30 and 60 nm in case of non-functionalised or fetuin-A-functionalised nanoparticles and between 100 and 200 nm in case of albumin-functionalised CaP nanoparticles. Each type of the characterised nanoparticles had a spherical shape after the initial synthesis. Note that the morphology of prepared CaP nanoparticles was investigated directly after the resuspension procedure. All nanoparticle preparations were stored in sterile water at 4°C and under these conditions, within 2 days the particles changed shape (or ‘ripened’) to needle-like shapes. The particles were not colloidally dispersed and therefore agglomerated. Particle solutions were vortexed immediately prior to addition to cells. All assays were performed with particles stored for at least 2 days and particles were checked for sterility using an endotoxin testing kit (stocks of particles had <0.5 EU/ml endotoxin, Pierce). The protein content of functionalised particles was determined by incubating particles in 0.1 M HCl for 30 minutes and measuring protein concentration using a BCA protein assay (Pierce). Stock solutions of CaP functionalised with fetuin-A (CaP/F) contained 88 µg/mL protein and 0.795 mg/mL Ca^2+^, and CaP functionalised with albumin (CaP/A) contained 205 µg/mL protein and 1.84 mg/mL Ca^2+^.

In separate experiments using soluble bovine fetuin-A and albumin (both cell culture suitable, Sigma) were prepared as stocks at 200 µM in water, filter sterilised, and stored at −20°C. Fetuin (Sigma) was confirmed to contain fetuin-A by Western analysis using an anti-fetuin-A rabbit polyclonal antibody kindly provided by Prof. W. Jahnen-Dechent, Aachen, Germany (data not shown). Human plasma-derived fetuin-A was used in some experiments and prepared as for bovine fetuin-A (Calbiochem).

### Toxicity Assay

An assay using propidium iodide (PI) as a measure of cell death was modified from Dengler WA *et al*, 1995 [21]. Briefly, cells were plated in 96-well plates at a density of 5,000 cells per well in medium containing serum. After an overnight incubation to allow adherence, cells were treated with or without CaP and/or various agents in serum-free M199 containing 1 µg/mL PI (1 mg/mL solution, Life Technologies). After 1 hour, the cells were washed 2 times with physiological buffer (see below) and PI uptake into dead cells was quantified using a Pherastar plate reader with optic module (peak excitation 520 nm, emission >610 nm). Autofluorescence was estimated in cells incubated as above but in the absence of PI. An average of the autofluorescence (blank) levels was subtracted from all samples for each experiment.

### Measurement of Intracellular Ca^2+^

Measurement of cytosolic Ca^2+^ was performed by monitoring fura-2 fluorescence of VSMCs adhered to glass coverslips using an Olympus Cell∧R imaging system. Human VSMCs were plated on glass coverslips at a density of 50,000 cells per well in a 12-well plate in culture medium supplemented with serum. The following day, the cells were loaded with 1 µM fura-2 acetoxymethyl ester (Life Technologies, 30 minutes incubation followed by a 30-minute period for de-esterification) in a freshly prepared physiological buffer containing essential amino acids (NaCl, 121 mM; KCl, 5.4 mM; MgCl_2_, 0.8 mM; CaCl_2_, 1.8 mM; NaHCO_3_, 6 mM; D-glucose, 5.5 mM; Hepes, 25 mM; essential amino acids (diluted from a 50×stock, PAA), pH 7.3). A single glass coverslip with adherent cells was mounted on the stage of the Cell∧R epi-fluorescence microscope in a temperature-controlled chamber (37°C). Fluorescence images were obtained with alternate excitation at 340 and 380 nm (with a 50 ms acquisition time and exposure every 3 s), while the emitted light was collected at 510 nm. Changes in fluorescence for individual cells were processed and analysed in Excel to generate ratio of emissions at the two excitation wavelengths, which is directly correlated to the amount of intracellular Ca^2+^. Calibration of intracellular Ca^2+^ levels was achieved as described previously [22].

### Cell Morphology and Imaging PI Uptake

Differential interference contrast (DIC) live cell imaging was performed in parallel to intracellular Ca^2+^ monitoring on the Cell∧R system to monitor cell morphology. In some experiments PI (1 µg/mL) was added to the extracellular solution at the start of the experiment and its uptake was monitored in parallel with fura-2 and DIC imaging. For detection of PI, cells were excited at 490 nm and the emitted light was collected at >620 nm. In some experiments, CaP particles were removed from VSMC cultures so that the morphology of underlying VSMCs could be observed. To remove CaP particles during VSMC culture, Ca^2+^ -free physiological buffer containing the complexing agent EGTA (4 mM, pH 7.3) was added to the cells.

### Electron Microscopy

VSMCs were incubated in 6-well plates at a density of 200,000 cells/well in growth medium. After 24 hours, cells were incubated in physiological buffer with or without CaP particles or fetuin-A, at 37°C. At specific time points, cells were rinsed briefly in 0.9% NaCl and fixed in 2% glutaraldehyde/2% formaldehyde in 0.05 M sodium cacodylate buffer at pH 8.0 at 4°C. Cells were removed from culture wells using cell scrapers and centrifuged at 10,000 rpm for 5 minutes. Fixed cells were resuspended in 0.05 M sodium cacodylate, pH 8.0. Osmium tetroxide buffered to pH 8.0 with sodium cacodylate was added to the cells for 1 hour. They were rinsed in distilled water, dehydrated in an ascending series of ethanol solutions and embedded in Quetol 651 epoxy resin. Sections were cut on a Rechert-Jung Ultracut S microtome, mounted on 400 mesh copper grids. Thin sections were examined by bright-field transmission electron microscopy (TEM) in an FEI Tecnai G2 operated at 120 kV. Images were recorded using an AMT XR60B camera running Deben software.

### Measurement of CaP Particle Dissolution

CaP particles (12.5 µg/mL) were incubated in either Ca^2+^-free physiological buffer at pH 7.3 or pH 6.0 in the presence or absence of fetuin-A. Incubations were carried out for 10 minutes at room temperature and then centrifuged at 13,000 rpm for 10 minutes. Supernatants were removed and CaP pellets were resuspended in 0.1 M HCl to dissolve particles. The Ca^2+^ content of the samples was measured by the cresolphthalein method, as described previously [23]. Particle morphology analysis was carried out after a 10-minute incubation in physiological buffer as detailed above and then immediately applied to 400 mesh carbon film grids. Grids were imaged using bright-field TEM as detailed in the ‘electron microscopy’ section above.

### Statistics

For PI assays, 4 replicate samples were used to determine the mean and S.D. for each treatment. Representative traces are shown for each experiment, with at least 3 different experiments using different human VSMC isolates showing similar results. Student’s t-test was used to test significance between two means.

A Fisher’s Exact test was performed to compare numbers of viable versus dead cells imaged for one hour, compared with cells from a separate session/treatment on the Cell∧R imaging system.

## Results

### Toxicity of CaP Particles is Reduced in the Presence of Fetuin-A or Albumin

**Figure 1:**
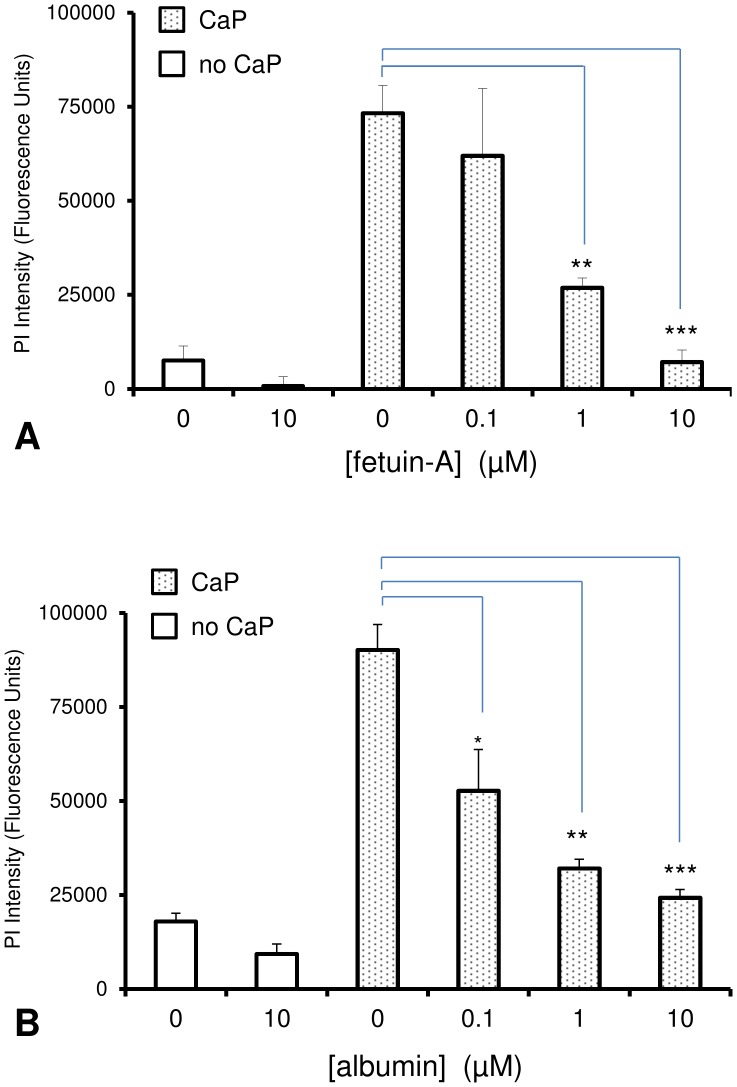
Effect of fetuin-A or albumin on CaP-induced VSMC death. VSMCs in 96-well plates were exposed to either control serum-free medium (no additions) or CaP particles (CaP, 25 µg/mL) in serum-free medium with or without different concentrations of fetuin-A (A) or albumin (B) for 1 hour. Both fetuin-A and albumin inhibited CaP-particle-induced VSMC death in a concentration-dependent manner, as measured by PI uptake (fluorescence intensity with blanks subtracted, *n* = 4, means ± S.D, *P<0.01, **P<0.0001, ***P<0.00001.).

We previously observed that foetal bovine serum reduced intracellular Ca^2+^ elevations and VSMC death after exposure to CaP particles. We hypothesised that CaP particle-binding proteins in serum, such as fetuin-A, could be responsible for this effect. To investigate if individual serum proteins could affect CaP toxicity, we tested the effects of bovine-derived fetuin-A and albumin on VSMC viability. Using PI influx as a measure of VSMC death, both fetuin-A and albumin reduced CaP-induced toxicity in VSMCs in a concentration-dependent manner (Fig. 1A and B).

### Fetuin-A and Albumin Inhibit Intracellular Ca^2+^ Elevations Induced by CaP Particles

**Figure 2:**
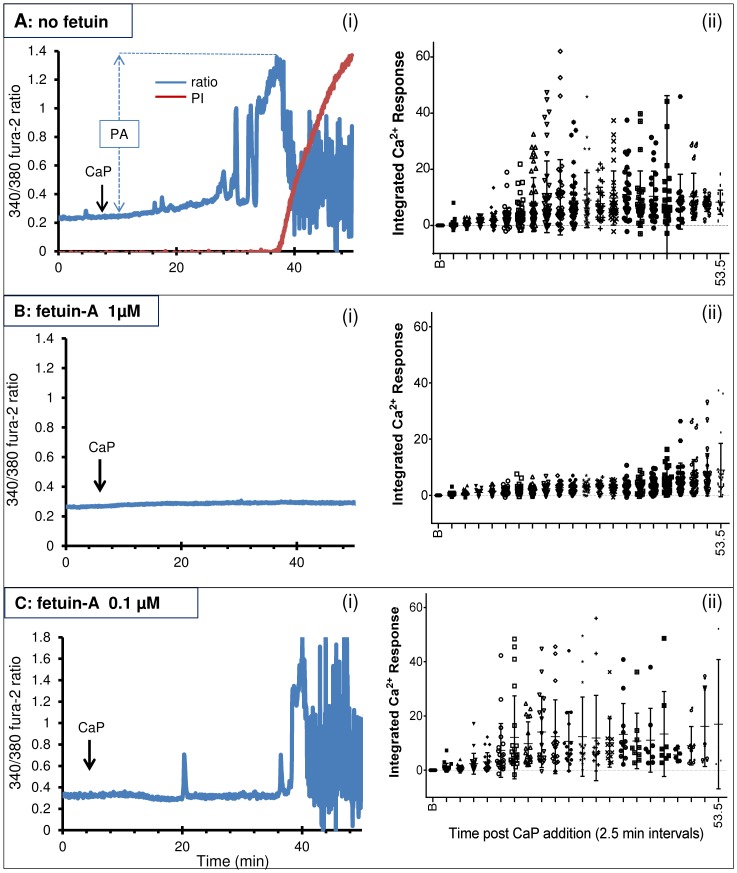
Effects of CaP particles on intracellular Ca^2+^ in the presence and absence of fetuin-A. Experiments were conducted either in the absence of fetuin-A (A), with 1 µM fetuin-A (B) or 0.1 µM fetuin-A (C). A-C(i) are representative traces showing intracellular Ca^2+^ changes in individual fura-2-loaded VSMCs on addition of 25 µg/mL CaP particles (arrow). A(i) Peak amplitude (PA) is indicated after addition of CaP particles. Note that fura-2 loss coincided with PI uptake, indicating time of cell death. Intracellular Ca^2+^ changes seen after cell death did not correspond to genuine Ca^2+^ signals (also explained in Fig. 3A and B). Incubation with 1 µM fetuin-A (B(i)) but not 0.1 µM fetuin-A (C(i)) silenced intracellular Ca^2+^ activity and prevented cell death. A-C(ii) Summary of intracellular Ca^2+^ activity in all VSMCs used for each condition. Each dot represents the area under the curve calculated for each 2.5 minute interval post CaP particle addition from individual cells. ‘B’ on the x-axis of these graphs represents the baseline, or 2.5 minutes prior to addition of CaP particles. These graphs display the range and magnitude of intracellular Ca^2+^ elevations after CaP particle addition from several individual experiments and also show that after addition of 1 µM fetuin-A, Ca^2+^ activity was vastly reduced and delayed (B(ii)).

We next addressed whether intracellular Ca^2+^ levels were affected by the presence of fetuin-A or albumin. In these studies, fura-2-loaded VSMCs were imaged by time-lapse video microscopy and intracellular Ca^2+^ and cell death were monitored in individual cells over one hour. Application of CaP particles evoked Ca^2+^ signals in cells, and initial elevations in intracellular Ca^2+^ appeared 14.6±1.3 min (mean ± s.e.m., *n* = 16) after CaP particle addition (Fig. 2Ai, representative graph and 2Aii). The magnitude and patterns of intracellular Ca^2+^ elevations differed in individual cells after exposure to particles (Fig. 2Aii). Cells that died exhibited a common substantial increase in intracellular Ca^2+^, just prior to cell death, which occurred at 31.3±1.76 min (*n* = 17) after CaP addition. Sometimes this was an abrupt Ca^2+^ elevation, but generally it was preceded by Ca^2+^ oscillations. Only those cells where intracellular Ca^2+^ levels increased to a particular threshold died (peak amplitude 0.93±0.03; *n = *17). VSMCs that survived exposure to CaP particles displayed smaller amplitude Ca^2+^ oscillations (peak amplitude 0.58±0.04; *n* = 8), compared with cells that died under the same conditions (P<0.0001). Calibration of the fura-2 ratio changes into Ca^2+^ concentration revealed that the CaP-induced Ca^2+^ oscillations had an average amplitude of 505 nM. Whereas, the large Ca^2+^ surge that immediately preceded cell death was 1520 nM. These data imply that CaP-induced elevations are not toxic unless a particular threshold is reached, causing Ca^2+^ overload and triggering cell death.

Despite the variation in CaP-induced Ca^2+^ signals generated by individual cells, clear differences in Ca^2+^ signals and time of cell death were observed in VSMCs exposed to CaP particles in the presence of fetuin-A or albumin. With fetuin-A at a concentration of 3 µM, intracellular Ca^2+^ elevations were either not observed or infrequent in cells treated with CaP particles and all cells survived (Fig. S1). With 1 µM fetuin-A, the response to CaP particles was smaller than in the absence of fetuin-A, and intracellular Ca^2+^ elevations were either absent or delayed (Fig. 2Bi and ii). With 0.1 µM fetuin-A, the magnitude and the timing of Ca^2+^ elevations observed in response to CaP particles were not significantly different from treatments with CaP particles alone (Fig. 2Ci and ii). We conclude that fetuin-A inhibits Ca^2+^ elevations and protects against cell death in a concentration-dependent manner. Similar results were obtained in experiments using albumin (summarised in Table 2 and Fig. S2). Human fetuin-A (1 µM) also reduced Ca^2+^ elevations and blocked cell death induced by CaP particles over a 1-hour period (Fig. S3).

**Table 2:** Cytotoxic effects of CaP particles in the presence or absence of fetuin-A or albumin or functionalised CaP particles (CaP with fetuin-A attached, CaP/F or with albumin attached, CaP/A) on fura-2 loaded VSMCs.

	Dead cells/total cells	% death	*n*
Control (no CaP)	0/80	0	15
CaP	82/102	80	17
CaP+fetuin-A (1 µM)	1/27	4	4
CaP+fetuin-A (0.1 µM)	19/22	86	4
CaP+albumin (1 µM)	1/21	5	4
CaP+albumin (0.1 µM)	18/23	78	4
CaP/F	9/49	18	8
CaP/A	14/16	88	3

Stock concentrations of particles contained 2.9 mg/mL Ca^2+^ for CaP, 0.8 mg/mL Ca^2+^ for CaP/F and 1.84 mg/mL Ca^2+^ for CaP/A. The particle concentration used in each experiment was 25 µg/mL in terms of Ca^2+^ content. Samples were prepared as a 250 µg/mL solution in 100 µl physiological buffer and added to VSMCs in a chamber containing 900 µl physiological buffer. All particle solutions were vortexed immediately prior to addition to cells. Raw data are presented, i.e. 82/102 denotes that 82 out of 102 cells that were imaged died within 1 hour of an experiment. Cell death was determined by fura-2 leak from cells. ‘*n*’ represents the number of separate experiments. Representative Ca^2+^ traces are shown in figure 2 and figures S2, S4 and S5.

We next examined whether CaP particles with fetuin-A or albumin specifically bound to them would affect VSMC viability. To achieve this, we synthesised CaP particles in the presence of fetuin-A or albumin, resulting in functionalised CaP particles that retained fetuin-A or albumin in aqueous solution. When CaP particles functionalised with fetuin-A (CaP/F) or albumin (CaP/A) were added to VSMCs, they reduced VSMC viability (Table 2). However, CaP/F was far less toxic than CaP/A or CaP particles, indicating that when bound to CaP, fetuin-A retained its cytoprotective effect, while albumin did not (Table 2 and Fig. S4 and S5). In addition, fetuin-A (1 µM) abolished the low levels of cell death induced by CaP/F particles (Fig. S6), suggesting that fetuin-A in solution may provide extra cytoprotective effects. Since fetuin-A bound to CaP particles inhibited cell death, we focussed on the mechanism of fetuin-A protection against VSMC death.

### Fetuin-A Acts Extracellularly to Protect against CaP-particle Induced Death

**Figure 3:**
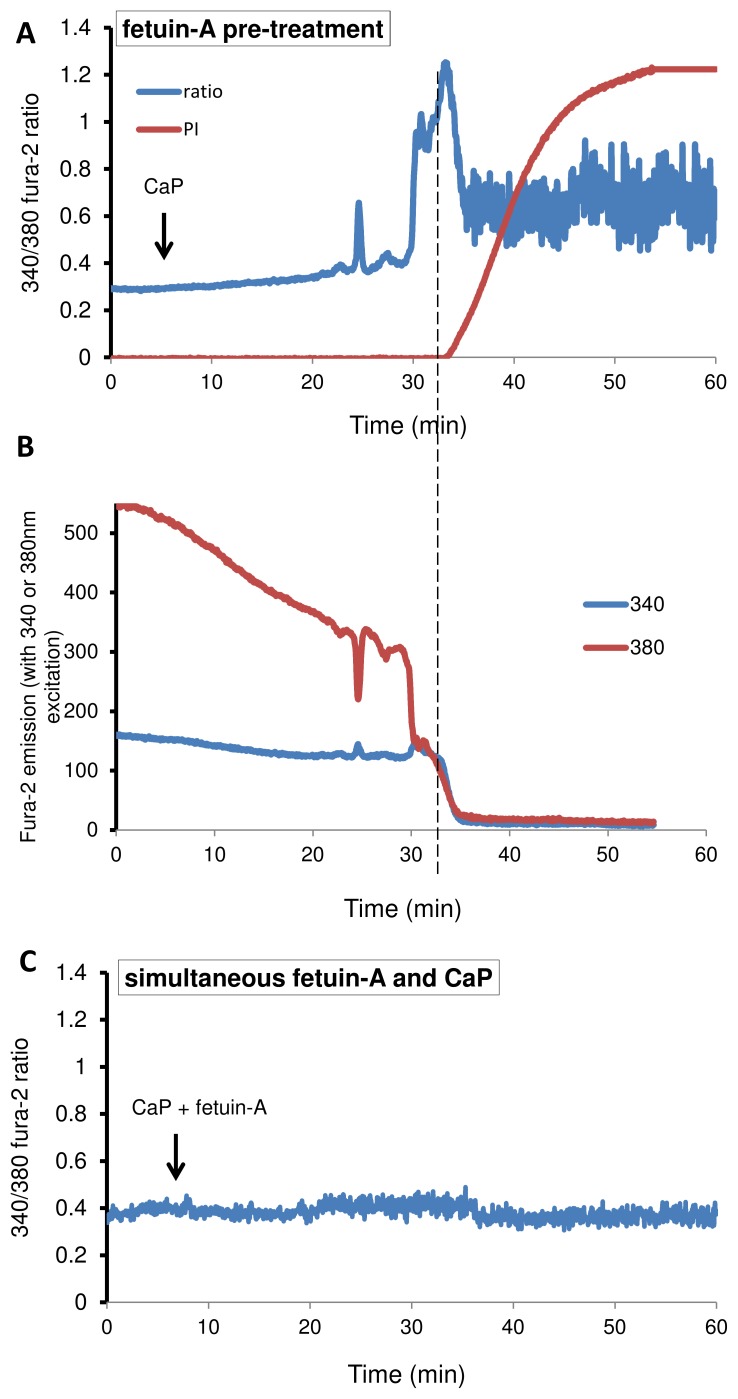
Timing of fetuin-A addition affects the response to CaP particles. Pre-incubating with fetuin-A (1 µM) for 15 minutes followed by replacing cell supernatant with fetuin-A-free physiological buffer gave no protection against cell death on CaP exposure (12.5 µg/mL) (A and B). The ratio of 340/380 fura-2 values is plotted in A and raw data is presented in B, showing that when 340 and 380 nm values both suddenly decline together (fura-2 leak from VSMC), this coincides with PI influx. The Ca^2+^ activity observed up to the time of fura-2 loss/PI influx represents genuine Ca^2+^ signals, whereas activity seen after this time point (in A) is not genuine. (C) Simultaneous addition of CaP (12.5 µg/mL) and fetuin-A (1 µM) had very little effect on VSMC intracellular Ca^2+^ and cells survived over one hour of analysis. Representative traces are shown.

Fetuin-A is known to bind CaP with high affinity and is therefore likely to alter the surface properties of CaP particles, thereby interfering with CaP particle exposure to the cell surface. Since fetuin-A is rapidly taken up by VSMCs and processed in endocytic vesicles, another possibility is that fetuin-A may act within the cell to dampen the effects of CaP particles or activate pro-survival signals. To test this, VSMCs were pre-incubated with fetuin-A, and the cell supernatant was then replaced with fetuin-A-free medium. The fetuin-A pre-treatment followed by removal of fetuin-A gave no protection against CaP particle-induced cell death, suggesting that fetuin-A uptake by VSMCs was not the mechanism for protection against CaP-induced cell death (Fig. 3A and B). In contrast, simultaneous addition of fetuin-A and CaP particles to VSMCs was sufficient to prevent Ca^2+^ signals and cell death, supporting the hypothesis that fetuin-A rapidly binds CaP particles (Fig. 3C). We concluded that fetuin-A was required to be present in the extracellular solution or bound to CaP particles in order to protect against CaP particle-induced cell death.

### CaP Particles Induce Plasma Membrane Damage and Large Blebs

**Figure 4:**
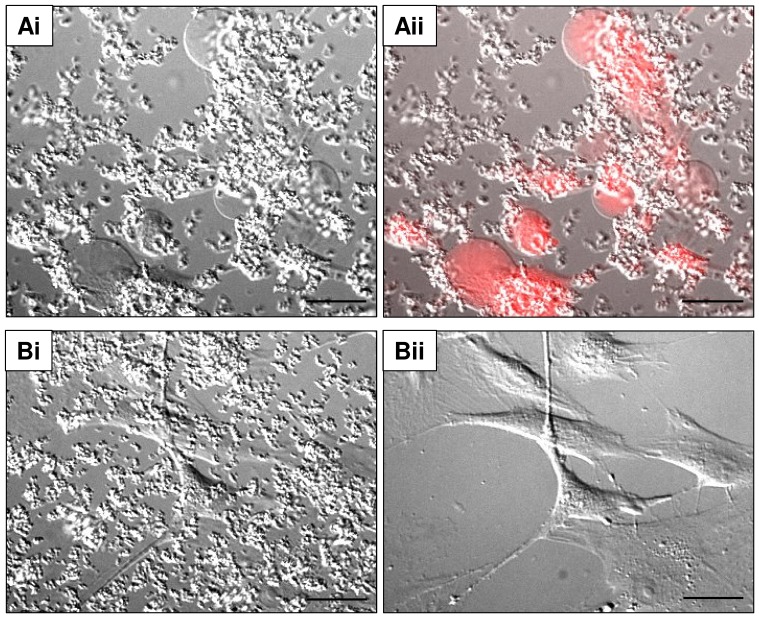
CaP particles induce bleb formation in human VSMCs. DIC images of VSMCs in physiological buffer after 1(25 µg/mL). CaP particles induced large bleb formation (Ai and ii) and these blebs contained PI (Aii). In the presence of fetuin-A (1 µM) and CaP particles (25 µg/mL), no blebs were seen (Bi and ii). After 1 hour of CaP and fetuin-A treatment, cells and particles in image Bi were treated with EGTA (4 mM) in Ca^2+^ -free physiological buffer to remove particles. After removal of particles, the morphology of underlying cells could be clearly observed (Bii). Scale bar: 50 µm.

To gain insight into the mechanism of cell death, we simultaneously monitored intracellular Ca^2+^ levels and the morphology of cells using high resolution DIC image capture. This latter technique revealed that agglomerated CaP particles settled rapidly on VSMCs, and that the cells consequently retracted and produced plasma membrane blebs that gradually expanded and protruded from the cell surface (Fig. 4Ai and Video S1). This type of large bleb formation is indicative of plasma membrane damage [24]. Some blebs retained PI for several minutes before the whole cell became PI positive, indicating that the damaged membrane was repaired/extruded via bleb release (Fig. 4Aii). In the presence of fetuin-A (≥1 µM), blebbing did not occur which supports the hypothesis that fetuin-A protected against CaP-induced cell damage (Fig. 4Bi and ii).

### Fetuin-A Delays CaP Particle Plasma Membrane Interactions and Damage

**Figure 5:**
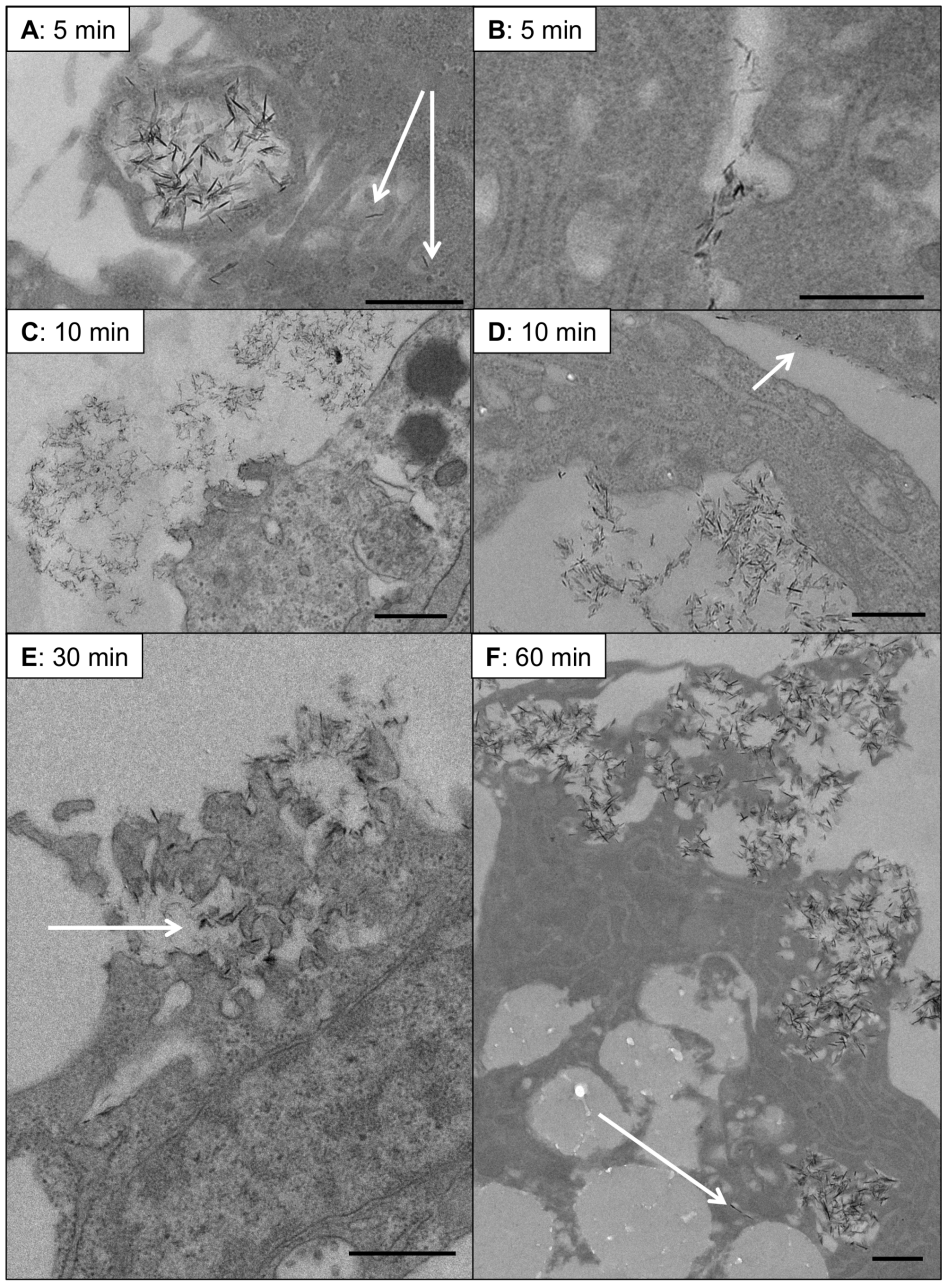
Ultrastructural analysis of CaP particle-exposure to VSMCs. VSMCs were incubated with CaP particles (12.5 µg/mL) for specific times (as indicated) before fixing and processing for TEM analysis. A. Evidence for macropinocytosis of clusters of CaP particles was often observed after 5 minutes of particle exposure and uptake of individual particles was also seen at this early timepoint (indicated by arrows in A). Clathrin-like pits were also often observed after CaP particle exposure (B). After 10 minutes of CaP particle exposure, plasma membrane damage was observed in association with membrane protrusions (C) or ingression (D). Discrete CaP particles were also seen aligning at the plasma membrane surface (arrow in D). After 30 minutes, areas of plasma membrane damage were observed and these areas contained electron-dense particles (indicated by arrow in E). B. At 60 minutes, intracellular particle accumulation in clusters or isolated particles (indicated by arrow) were observed and large areas of plasma membrane rupture (F). Bar = 500 nm.

**Figure 6:**
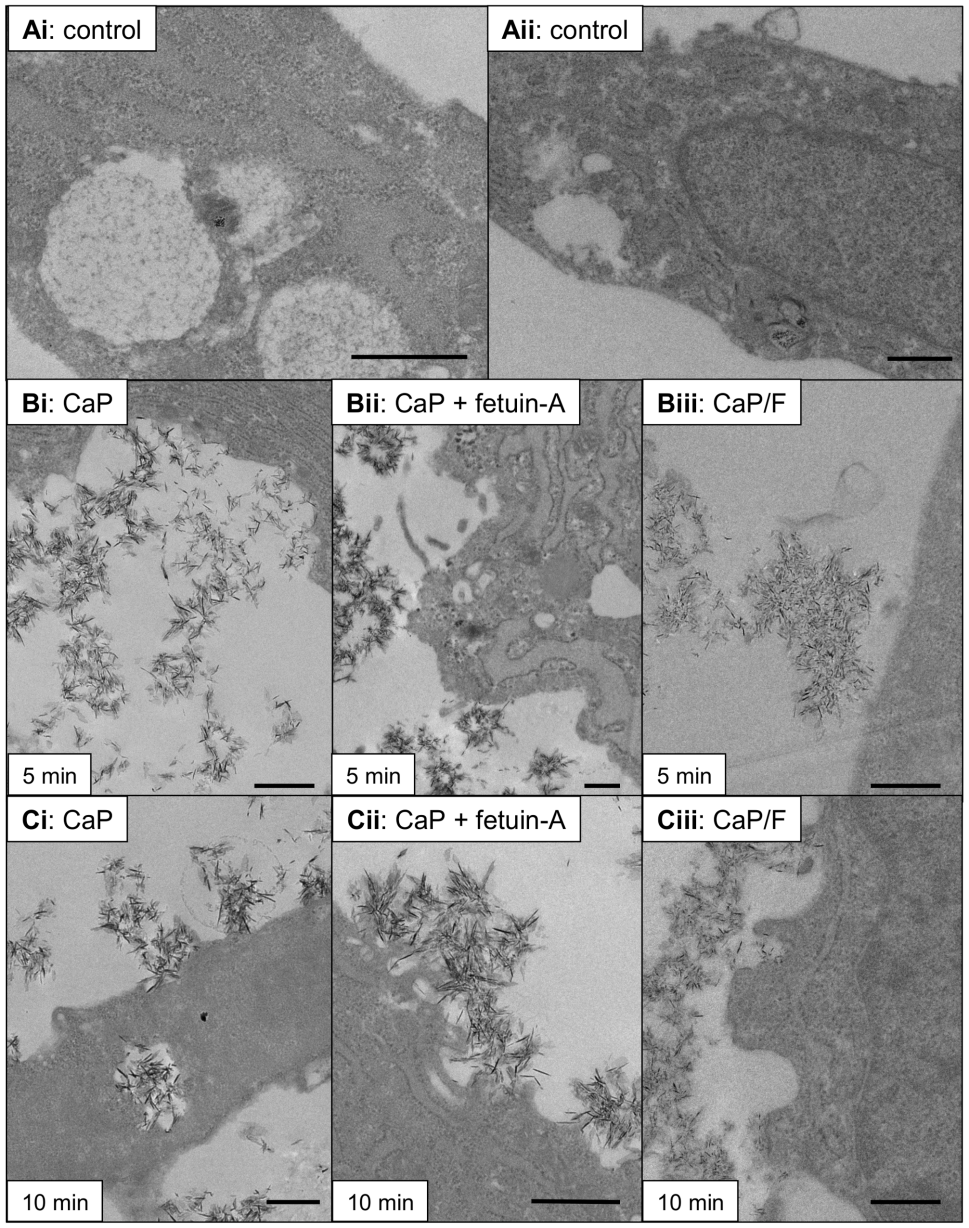
TEM images of CaP particle/VSMC interactions in the presence of fetuin-A. TEM images show ultrastructural features observed after treatment of VSMCs with CaP particles (12.5 µg/mL) ± fetuin-A (1 µM) or with CaP/F (12.5 µg/mL). Control images from cells exposed to physiological buffer without CaP particles are shown in Ai and Aii. After 5 minutes of particle exposure (middle panel), CaP/plasma membrane interactions were observed (Bi) but these interactions were not commonly observed in the presence of fetuin-A (Bii) or with CaP/F particles (Biii). After 10 minutes (lower panel, Ci–iii), CaP particle interactions with the plasma membrane were detected for each condition. Bar = 500 nm.

Transmission electron microscopy (TEM) analysis revealed that CaP particles interact with the VSMC plasma membrane and are taken up into VSMCs as early as 5 minutes after addition of particles (Fig. 5). Macropinocytosis of clusters of particles was observed as well as uptake via plasma membrane invagination or incorporation of individual particles into cells (Fig. 5A and B). Focal plasma membrane damage was also commonly observed after 5 or 10 minutes of particle exposure. Damage at the plasma membrane was often associated with clusters of particles and cellular protrusions (Fig 5C), but clusters of particles were also observed in areas of the cell that appeared to be eroded or retracting away from the subjacent particles (Fig. 5D). In addition, individual particles were often seen either bound to the plasma membrane surface or entering the cell with no apparent damage after 10 minutes of particle exposure (Fig. 5C). Thus, at early time points, CaP particles appeared to interact with VSMCs in various ways. Profound plasma membrane damage was seen in association with clusters of CaP particles after 30 and 60 minutes of addition of particles (Fig. 5E and F). Within cells, individual particles were detected as well as clusters of particles within large cellular compartments or ‘vesicles’ (Fig. 5F and Fig. 6Ci). From these observations we postulate that CaP particles can both bind to the VSMC plasma membrane surface and enter VSMCs via different mechanisms. Our TEM analysis indicates that focal damage to VSMCs occurs within 5–10 minutes of exposure to CaP particles. However, the imaging studies described above indicated that loss of membrane integrity and cell death occurred much later (approximately 30 minutes, Fig. 3A and B). We postulate that the early membrane damage is localised at the site of CaP particle/plasma membrane interaction and does not disrupt cellular homeostasis. The focal interaction of CaP particles with membranes may activate repair mechanisms by promoting a limited influx of Ca^2+^. This CaP-induced Ca^2+^ entry may be a component of the Ca^2+^ oscillations triggered by CaP addition (Fig. 2). However, with continued exposure to CaP particles, repair and intracellular Ca^2+^ homeostatic mechanisms become overwhelmed because of Ca^2+^ overload, and large plasma membrane blebs are formed as a final attempt to rescue cell integrity.

**Figure 7:**
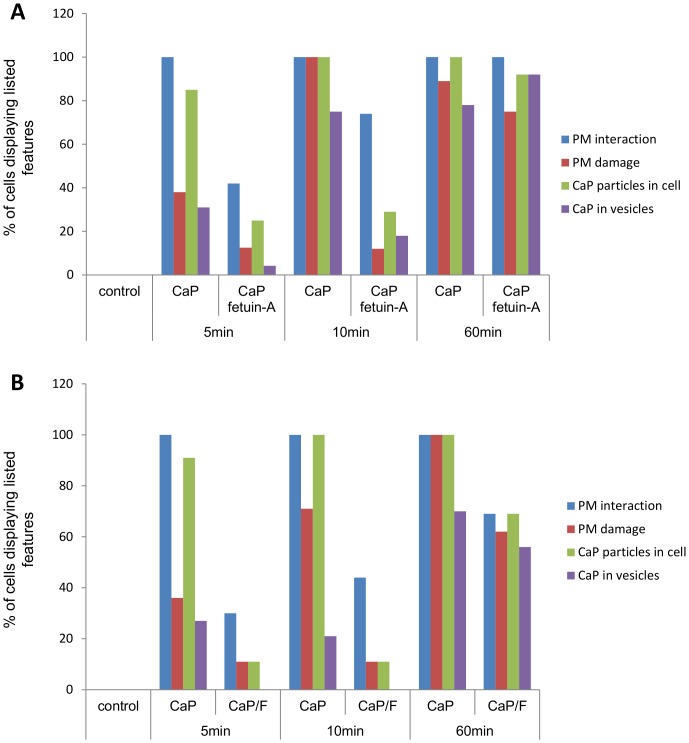
TEM analysis of CaP particle/VSMC interactions in the presence of fetuin-A. A. Images taken at random (see Figure 6) were assessed for various features including: VSMC plasma membrane interaction; plasma membrane damage; CaP particles seen within cells and CaP particles seen within discrete intracellular compartments (vesicles) (*n* ≥9 for each condition/time point). Controls were samples exposed to serum-free physiological buffer without particles. A clear trend was observed where the presence of fetuin-A reduced CaP interactions with cells up to 10 minutes of exposure. However, the presence of fetuin-A did not appear to influence CaP interactions/uptake after 60 minutes of exposure. B. As for A, but the effect of CaP particles on VSMC interactions/uptake was compared with CaP/F (*n* ≥9). As for fetuin-A added in solution (A), the presence of fetuin-A on CaP particles appeared to delay early interactions with VSMC.

To investigate whether fetuin-A could affect CaP particle interaction with VSMCs we used TEM analysis at different time points after CaP particle exposure (Fig. 6). After 5 minutes, fewer CaP particle-plasma membrane interactions were observed in the presence of fetuin-A (1 µM) (Fig. 6Bi and Bii). Similarly, functionalised CaP/F particles displayed a delay in interaction with VSMCs compared with non-functionalised CaP particles at this early time point (Fig. 6Bi and Biii). After 10 minutes of exposure to CaP/F or CaP+fetuin-A, a greater number of cells displayed CaP-plasma membrane interactions and uptake of CaP particles (Fig. 6C), but the percentage of cells displaying these interactions was still lower compared with CaP particles alone (Fig. 7A and B). The delay in interaction of CaP particles with VSMCs in the presence of fetuin-A was likely to contribute to its cytoprotective effects. However, after 60 minutes of exposure of VSMCs to CaP particles, there was no distinct difference in TEM features between cells treated with CaP alone, CaP/F or CaP+fetuin-A (Fig. 7A and B). These data indicate that fetuin-A delays the membrane-damaging effects of CaP on VSMCs, but does not persistently inhibit CaP-VSMC interactions. Thus, the majority of VSMCs exposed to CaP particles in the presence of fetuin-A contained intracellular particles after 60 minutes.

### Effects of Fetuin-A on CaP Particle Dissolution

The results described above present an interesting paradox. Our imaging studies and cell death assays suggested that fetuin-A protected cells against CaP crystals for >60 minutes. In particular, with CaP+fetuin-A, we did not observe the high amplitude Ca^2+^ signals that are indicative of disasterous homeostatic loss. TEM analysis, however, indicated that the degree of CaP-VSMC interaction and CaP engulfment was the same at 60 minutes. A plausible explanation for these apparently contradictory observations is that in addition to delaying CaP-membrane interaction, fetuin-A reduces the toxicity of CaP particles when they are inside VSMCs.

**Figure 8:**
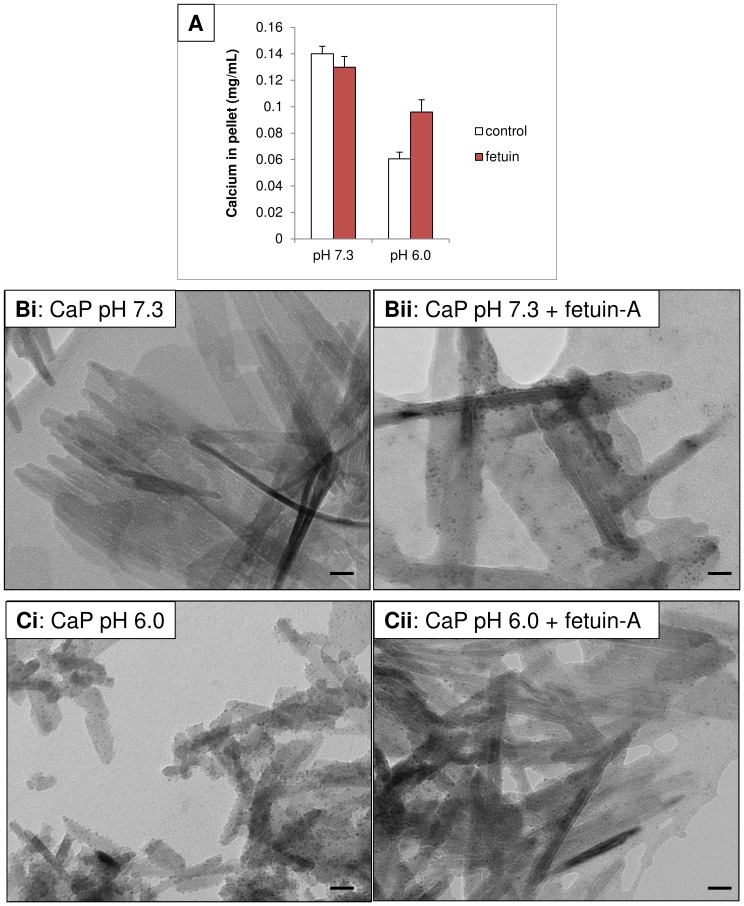
Fetuin-A inhibits CaP dissolution. CaP particles (12.5 µg/mL) ± fetuin-A (1 µM) were exposed to Ca^2+^-free physiological buffer, either pH 7.3 or pH 6.0 for 10 minutes at room temperature. A. CaP particles were pelleted by centrifugation, resuspended in 0.1M HCl and Ca^2+^ was measured using the cresolphthalein method. CaP particles did not dissolve at pH 7.3, but partial dissolution was observed at pH 6.0. Dissolution at pH 6.0 was inhibited in the presence of fetuin-A (*n* = 3, mean ± S.D.). B. TEM analysis of CaP particles exposed to physiological buffer at pH 7.3 (i) or in the presence of fetuin-A (ii). C. TEM analysis of CaP particles exposed to physiological buffer at pH 6.0 (i) or in the presence of fetuin-A (ii). Bar = 20nm.

To investigate why intracellular CaP particles are less toxic when fetuin-A is present, we tested whether fetuin-A could affect CaP particle dissolution. We attempted to mimic intra-endosomal/lysosomal acidic conditions and to examine the effects of fetuin-A on particle dissolution at neutral or acidic pH. Using a calcium assay to estimate dissolved Ca^2+^ from CaP particles, we found that CaP particle dissolution occurred in physiological buffer at pH 6.0 (Fig. 8A). However, in the presence of fetuin-A (1 µM), CaP particle dissolution was inhibited. TEM analysis of CaP particles exposed to physiological buffer at pH 6.0 revealed that particles had an altered morphology, appeared partially fragmented and were smaller than CaP particles in physiological buffer at pH 7.3 (Fig. 8Bi and Ci). However, fetuin-A treated CaP particles in physiological buffer at pH 6.0 retained their elongated, needle-like morphology, implying that fetuin-A protected from dissolution (Fig. 8Cii). This is consistent with the hypothesis that fetuin-A stabilises CaP particles and slows their dissolution within intracellular acidic vesicles. The reduction in CaP dissolution would thereby prevent or delay cytotoxic Ca^2+^ elevations arising inside the cells.

## Discussion

Here, we tested whether the CaP crystal-binding plasma proteins fetuin-A and albumin could affect the toxicity of CaP particles. We found that both proteins inhibited elevations in intracellular Ca^2+^ and reduced cell death during exposure to CaP particles in a concentration-dependent manner. Although the Ca^2+^ elevations in individual cells displayed different patterns, a large unrecoverable rise in intracellular Ca^2+^ occurred in all cells that died, suggesting that high levels of Ca^2+^ could not be tolerated, resulting in necrosis. In cells that survived CaP particle treatment, their intracellular Ca^2+^ oscillations had lower amplitudes indicating that all cells reacted to CaP exposure in terms of intracellular Ca^2+^ elevations but intrinsic heterogeneity of Ca^2+^ homeostatic mechanisms may be responsible for the observed differences in survival. Fetuin-A and albumin reduced both toxic and non-toxic intracellular Ca^2+^ responses to CaP particles, suggesting that these proteins may block or delay interactions of CaP particles with VSMCs.

Levels of fetuin-A or albumin that are normally found in the circulation (approximately 10 µM) vastly reduced CaP particle-induced cell death. At lower concentrations of fetuin-A or albumin (≥1 µM) a reduction and delay in Ca^2+^ signals was observed and cell death was inhibited, while 0.1 µM fetuin-A or albumin afforded no protection against CaP particles. This suggests that if levels of fetuin-A or albumin are reduced, they may no longer protect against CaP particle-induced intracellular Ca^2+^ elevations or cell death. Indeed, although fetuin-A appears to be abundant in the circulation, levels of fetuin-A in healthy, calcification-free arteries have been reported to be very low or absent, and other calcification inhibitors such as matrix Gla protein produced by VSMCs are thought to actively inhibit CaP crystal formation in arteries [25], [26]. Fetuin-A is synthesised in the liver and via the circulation it accumulates in bone and in calcified arteries and therefore appears to be sequestered in areas of physiological and pathological calcification. Fetuin-A has potent anti-calcifying activity, but much less is known about albumin-CaP interactions. It is thought that when calcification occurs, fetuin-A and albumin bind newly formed crystals and prevent them from developing further and promote their removal via scavenger receptors on phagocytic cells [16]. This mechanism of inhibition of calcification is thought to contribute to the maintenance of calcification-free tissues and is likely to be less efficient if levels of fetuin-A and albumin are reduced. Indeed, circulating levels of fetuin-A and albumin are reduced in CKD patients [12], [15], [27] and one study reports that albumin levels decrease with ageing [28]. Thus reduced levels of circulating fetuin-A and albumin in patients appear to be associated with excessive vascular calcification.

To test the impact of bound versus free fetuin-A and albumin, CaP particles were synthesised in the presence of these proteins to create particles with a protein ‘corona’. Clearly, fetuin-A functionalised particles (CaP/F) were less toxic than naked particles, implying that the fetuin-A component of the particles reduced their toxicity. It was interesting that relatively low levels of cell death occurred with CaP/F particles and that this toxicity was abolished by incubation with soluble fetuin-A. This suggests that fetuin-A may have additional cytoprotective effects. The toxicity of albumin-functionalised particles (CaP/A) was comparable to naked particles. The reason for the lack of protection by albumin in functionalised form is not clear, but we speculate that albumin is not as potent as fetuin-A when bound to CaP particles. As fetuin-A inhibited cell death in particle-bound form, we focussed on how fetuin-A achieved this and how CaP particles interact with VSMCs.

Our data suggest that the mechanism of CaP particle-induced Ca^2+^ elevations and cell death may involve more than one pathway. TEM studies confirmed that CaP particles were found within cells, some of which were located to intracellular vesicles, which supports our previous studies that lysosomal acidification is involved in CaP particle-induced Ca^2+^ elevations and cell death [8]. We also observed that some particles entered cells as individual particles with no apparent damage to the plasma membrane and no evident vesicle association. The fate of these individual particles within VSMCs is not yet known. Furthermore, we observed plasma membrane damage, typically associated with clusters of CaP particles. The morphology of the membrane at sites of damage was particularly interesting, either with cellular protrusions or clefts displaying interactions with the particles. These sites show extreme invagination of the plasma membrane with similarities to those seen at the membrane of striated muscle at the sites of neuromuscular junctions. Plasma membrane damage has been described for other types of nanoparticles but to our knowledge, this is the first description of this type of membrane protrusion/particle interaction. In human erythrocytes, binding of monosodium urate or calcium pyrophosphate dihydrate particles to membranes induces redistribution of transmembrane proteins leading to ‘pore’ formation [29]. Ca^2+^ entry via plasma membrane damage has been shown in other studies to activate repair mechanisms involving annexins and subsets of lysosomes [30], [31]. The finding of isolated PI-rich blebs indicated that PI entered via damaged membrane sites but that the membrane was rapidly repaired and the damaged membrane was extruded from the cells. With continued exposure to CaP particles, constant stimulation of repair and calcium homeostatic mechanisms is likely to become dysfunctional, resulting in Ca^2+^ overload, large bleb formation as a final attempt to rescue cell integrity and finally cell death.

The cytoprotective effect of fetuin-A may be attributed to binding CaP particles, thereby delaying plasma membrane interactions and damage. In addition, fetuin-A protected cells from the toxic effects of CaP following their entry into cells. With regard to the latter effect, a plausible mechanism was proposed by Smith *et al* [32] where they speculated that fetuin-A could inhibit lysosomal dissolution of CaP particles within acidic organelles such as lysosomes, consequently slowing the release of Ca^2+^ to the cytosol. In studies in HeLa cells, CaP particles conjugated with the pH indicator SNARF-1 were shown to be endocytosed and incorporated into lysosomes, detected by a switch from neutral to acidic pH on particles [33]. We demonstrated that fetuin-A slowed the dissolution of CaP particles under acidic conditions which may explain why VSMCs survived in the presence of fetuin-A, despite the presence of numerous intracellular CaP particles. TEM analysis detected clusters of CaP particles in discrete compartments within VSMCs but whether CaP particles bound to fetuin-A enter endosomes or lysosomes in VSMCs is yet to be determined.

The observed delay in uptake of CaP particles in the presence of fetuin-A contradicts previous literature suggesting that fetuin-A is an opsonin, facilitating phagocytosis. Indeed, VSMCs are efficient phagocytes in terms of apoptotic cell phagocytosis [34]. However, the mechanism of uptake of CaP particles by VSMCs may not involve classic receptor-mediated phagocytosis. We demonstrated evidence for macropinocytosis, plasma membrane invagination resembling clathrin-mediated endocytosis and also uptake of individual particles with no evident plasma membrane disruption. Particle charge is likely to be important in CaP particle uptake. CaP particles have a net weak positive charge, which could explain their affinity for the negatively charged phospholipids or glycosaminoglycans on the plasma membrane surface. Fetuin-A and albumin are negatively charged at pH 7, and by binding CaP particles they are expected to change the particle net charge to negative. Alteration of crystal charge may account for the delayed cellular uptake and reduced plasma membrane damage of CaP particles in the presence of fetuin-A. In studies using modified polystyrene nanoparticles and fluorescently labelled serum, the entry of these particles into human cell lines was described as a ‘Trojan horse’ effect [35]. The authors suggested that the particle corona protects membranes from initial damage upon entry into cells but that when particles accumulate in lysosomes, the corona is degraded, exposing the bare particle surface. This is thought to cause lysosomal damage followed by apoptosis, which has also been observed in studies using cholesterol nanocrystals [7]. These studies suggest that it is only a matter of time before the protein corona is removed and the damaging particle surface is exposed within the cell. In our previous studies where CaP particles were exposed to VSMCs in the presence of serum, VSMC apoptosis was stimulated after 24 hours [8]. It would therefore be valuable to investigate the timing of fetuin-A disassociation from CaP particles inside cells and to determine the impact on lysosomal integrity and cell viability.

Other studies have shown that crystalline material may interact with various circulating proteins, including albumin, fetuin-A, fibrin, fibronectin, transferrin, acute phase proteins and lipoproteins [17], [36]. Alterations in shape, size or charge may modulate particle interactions with cells, including kinetics of phagocytosis and toxicity, as well as modify crystal-induced inflammation [18], [37]. It is assumed that CaP crystals at all extracellular sites *in vivo* will be coated with proteins as CaP has a high affinity to biomolecules. However, it is possible that bare patches of exposed crystal may occur. Positron emission tomography (PET) scanning and 18F-sodium fluoride have been used to detect new bone formation in patients, a technique that detects F replacement of OH in newly formed hydroxyapatite. This technique has been used for several years to study bone formation and in detection of calcification in tumours. It has recently been developed to detect active calcification in arteries as a potential marker of unstable atherosclerotic plaques [38]. Evidence from PET scanning suggests that at least some of the CaP particles present *in vivo* have an exposed surface representing newly formed CaP crystals or osteoclastic activity on established calcification. From our *in vitro* studies, we postulate that CaP particles with an exposed surface are more toxic to VSMCs than CaP particles bound to proteins such as fetuin-A and albumin. CaP particles will come in contact with various proteins but fetuin-A may have stronger binding to CaP, compared with other proteins. Our studies are therefore most relevant to situations where fetuin-A levels are low, as described above, and also where bare CaP particles are exposed such as pathological situations in which excessive production of crystals is accompanied by low levels of clearance or areas of osteoclastic or protease activity in atherosclerotic plaques. Whether other closely interacting factors such as cholesterol, glycosaminoglycans or other proteins can influence CaP particle activity is yet to be determined.

Specific phenotypes of VSMCs may be particularly susceptible to CaP-induced cell death, since some VSMCs survived CaP particle treatment. Other cell types such as fibroblasts are stimulated to proliferate when exposed to similar levels of CaP particles [39]. In macrophages and chondrocytes, CaP particles have been shown to induce apoptotic cell death [18], [40]. In serum-free conditions similar to those used in our study, human monocyte-derived macrophages responded to CaP particles by inducing formation of a large plasma membrane compartment that sequesters nanoparticles from the extracellular space without cellular uptake [41]. The particles are then processed to the cytosol and degraded gradually, lessening their toxic potential. Dispersion of agglomerated CaP particles appeared to reduce particle uptake in human macrophages [42]. From our TEM studies, agglomerates or clusters of particles appeared to be damaging in VSMCs. In other studies where colloidal dispersions of CaP nanoparticles have been used, there were no adverse effects on cells [43]. Thus the toxicity of CaP particles depends on several factors including size, charge, particle-associated factors, degree of dispersion, target cell phenotype and time of exposure.

## Conclusions

In conclusion, we found that CaP particles induced cell death in VSMCs, and that this involved progressive plasma membrane damage and cellular uptake of particles. Plasma membrane interaction and the observations of variously sized groups of CaP particles within cells suggest that several different mechanisms are involved. Both plasma membrane damage and uptake of CaP particles contribute to intracellular Ca^2+^ elevations that eventually overwhelm repair and homeostatic mechanisms causing cell death. Our results suggest that fetuin-A protects VSMCs from the cytotoxic effects of CaP by binding to CaP particles, delaying their interaction with the VSMC plasma membrane, delaying their accumulation in VSMCs and by stabilising the particles, thereby slowing their intracellular dissolution. Thus, the binding of fetuin-A to CaP particles renders them less harmful to VSMCs and is expected to dampen the pro-inflammatory and pro-calcification effects of damaged VSMCs. These studies are particularly relevant to situations where fetuin-A and albumin levels are low and where new crystal formation outweighs calcification-inhibitory mechanisms. Since an extracellular source of fetuin-A is required to inhibit CaP particle toxicity, this may be beneficial in therapeutic applications, not only in patients with severe calcification but also in designing biomaterials with reduced cytotoxic potential.

## Supporting Information

Figure S1 **Effects of CaP particles on intracellular Ca^2+^ in the presence of fetuin-A (3 µM).** A and B are representative traces showing a lack of response to CaP (A) or non-toxic intracellular Ca^2+^ changes (B) in individual fura-2-loaded VSMCs on addition of 25 µg/mL CaP particles (arrow indicates time of addition) in the presence of 3 µM fetuin-A. Cell death was not observed in the presence of 3 µM fetuin-A over 1 hour of analysis.
(TIF)

Figure S2 **Effects of CaP particles on intracellular Ca^2+^ in the presence albumin (1 µM).** A and B are representative traces showing intracellular Ca^2+^ changes in individual fura-2-loaded VSMCs on addition of 25 µg/mL CaP particles (arrow indicates time of addition) in the presence albumin (1 µM). Cells displayed either no intracellular Ca^2+^ changes (A) or clear intracellular Ca^2+^ spikes (B) over 1-hour of analysis. Under these conditions 1 out of 21 cells died (also detailed in Table 2).
(TIF)

Figure S3 **Effects of CaP particles on intracellular Ca^2+^ in the presence of human fetuin-A (1 µM).** A and B are representative traces showing intracellular Ca^2+^ changes in individual fura-2-loaded VSMCs on addition of 25 µg/mL CaP particles (arrow indicates time of addition**)** in the presence of human fetuin-A (1 µM). Cells displayed either no intracellular Ca^2+^ changes (A) or clear intracellular Ca^2+^ spikes (B) over 1 hour of analysis. Under these conditions no cells died.
(TIF)

Figure S4 **Effects of functionalised CaP/F particles on intracellular Ca^2+^.** A and B are representative traces showing intracellular Ca^2+^ changes in individual fura-2-loaded VSMCs on addition of 25 µg/mL CaP/F particles (arrow indicates time of addition). All cells displayed intracellular Ca^2+^ changes in response to CaP/F particles over 1 hour of analysis. These intracellular Ca^2+^ responses were either non-toxic (40 out of 49 cells, 82% as in A) or toxic (9 out of 49 cells, as in B, also detailed in Table 2).
(TIF)

Figure S5 **Effects of functionalised CaP/A particles on intracellular Ca^2+^.** A and B are representative traces showing intracellular Ca^2+^ changes in individual fura-2-loaded VSMCs on addition of 25 µg/mL CaP/A particles (arrow indicates time of addition). All cells displayed intracellular Ca^2+^ changes in response to CaP/A particles over 1 hour of analysis. These intracellular Ca^2+^ responses were either non-toxic (2 out of 16 cells, 12% as in A) or toxic (14 out of 16 cells, 88%, as in B, also detailed in Table 2).
(TIF)

Figure S6 **Effects of functionalised CaP/F particles on intracellular Ca^2+^ in the presence of fetuin-A (1 µM).** A and B are representative traces showing intracellular Ca^2+^ changes in individual fura-2-loaded VSMCs on addition of 25 µg/mL CaP/F particles (arrow indicates time of addition) in the presence of fetuin-A (1 µM). No cells died with this treatment over 1 hour of analysis. Two different types of intracellular Ca^2+^ responses were observed, either no response (A), or cells displaying small changes in intracellular Ca^2+^ (B).
(TIF)

Video S1 **CaP particles induce bleb formation in human VSMCs.** Video showing DIC images of VSMCs in physiological buffer with live addition of CaP particles (25 µg/mL) over 1 hour of imaging.
(AVI)
