# Supplementary figures and images for: Fetuin-A and Albumin Alter Cytotoxic Effects of Calcium Phosphate Nanoparticles on Human Vascular Smooth Muscle Cells
Source: PLoS One. 2014 May 21;9(5):e97565. doi: 10.1371/journal.pone.0097565 (PMC4029753; doi:10.1371/journal.pone.0097565)

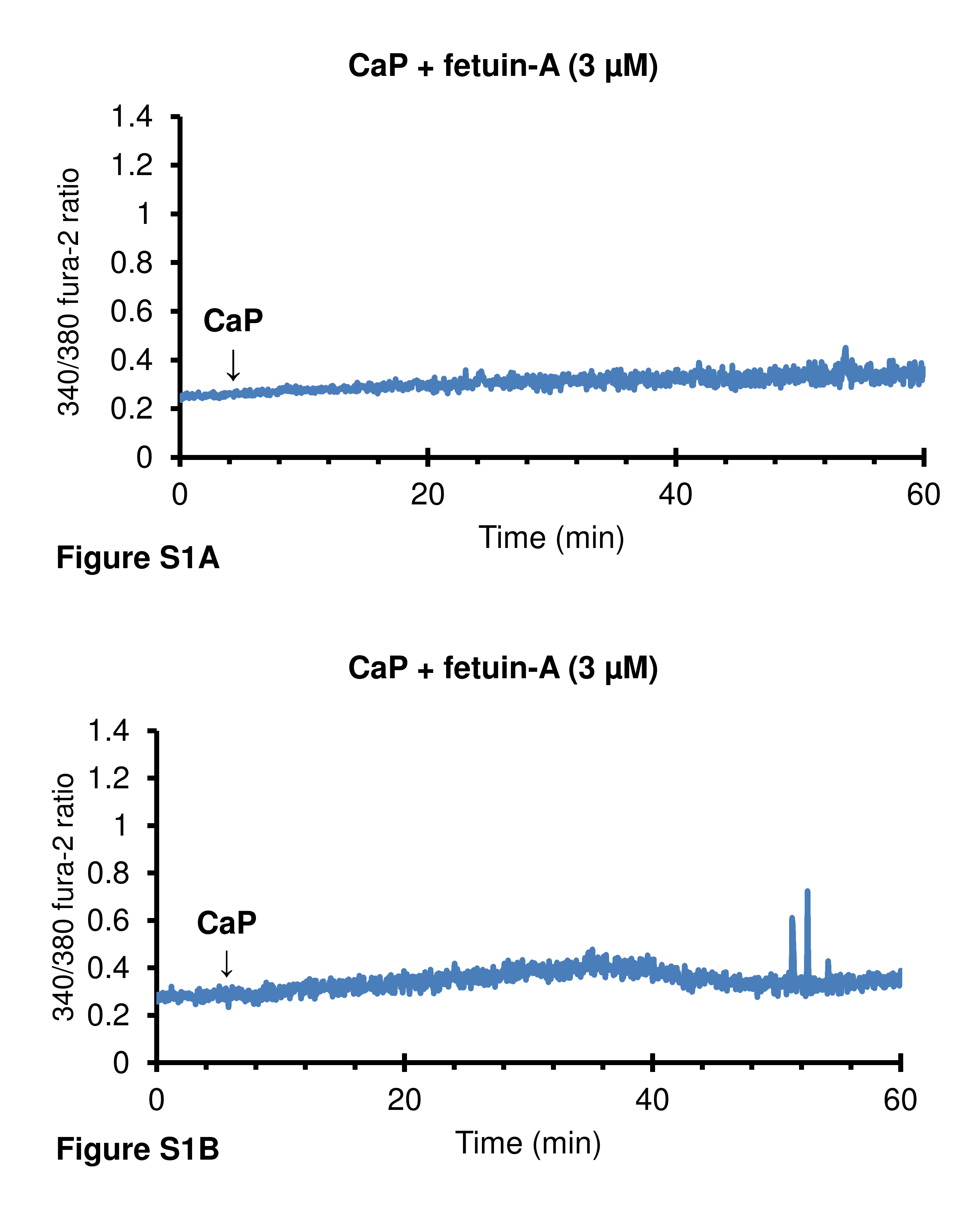

Supplement: Figure S1 — Effects of CaP particles on intracellular Ca2+ in the presence of fetuin-A (3 µM). A and B are representative traces showing a lack of response to CaP (A) or non-toxic intracellular Ca2+ changes (B) in individual fura-2-loaded VSMCs on addition of 25 µg/mL CaP particles (arrow indicates time of addition) in the presence of 3 µM fetuin-A. Cell death was not observed in the presence of 3 µM fetuin-A over 1 hour of analysis. (TIF) [file pone.0097565.s001.tif]

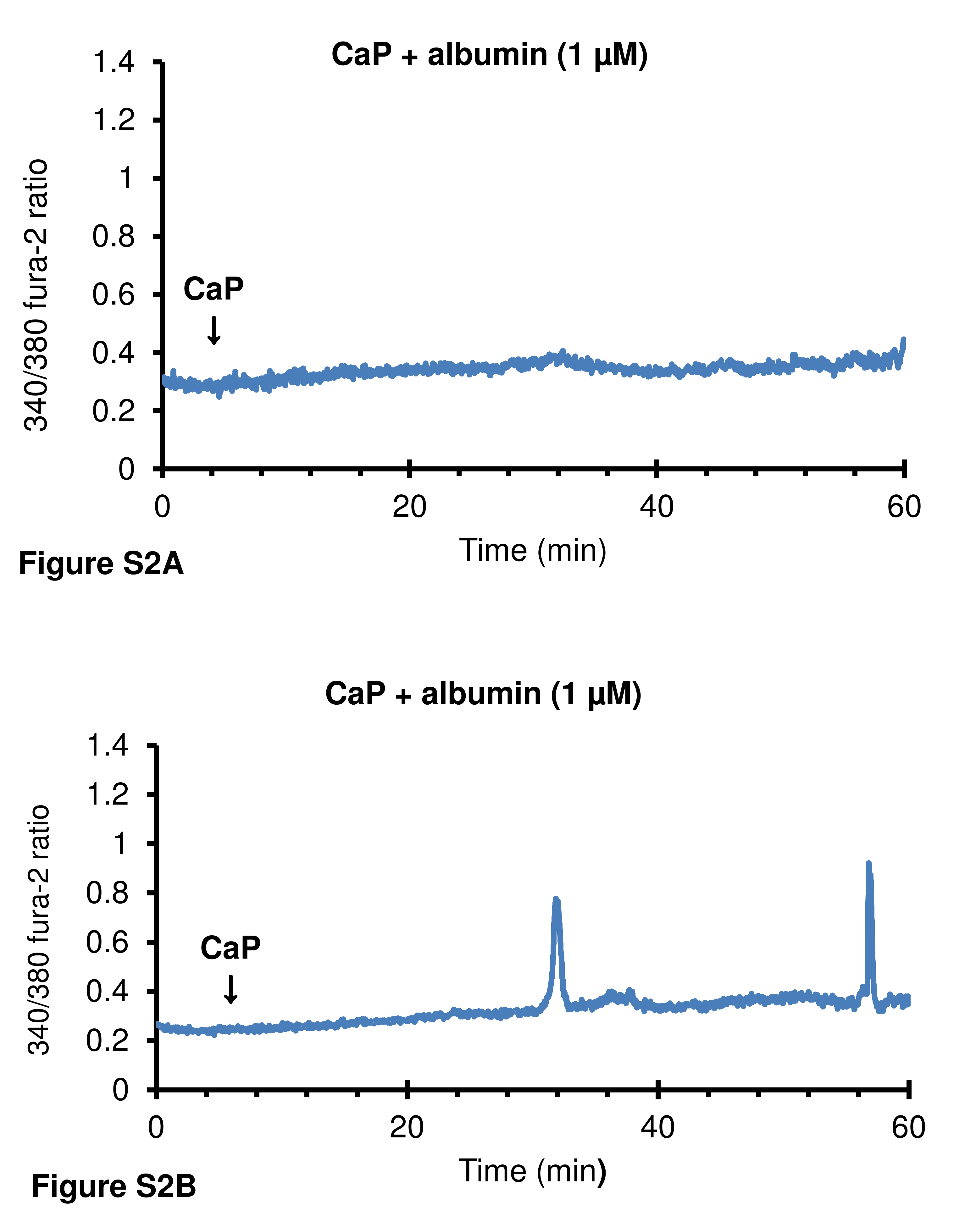

Supplement: Figure S2 — Effects of CaP particles on intracellular Ca2+ in the presence albumin (1 µM). A and B are representative traces showing intracellular Ca2+ changes in individual fura-2-loaded VSMCs on addition of 25 µg/mL CaP particles (arrow indicates time of addition) in the presence albumin (1 µM). Cells displayed either no intracellular Ca2+ changes (A) or clear intracellular Ca2+ spikes (B) over 1-hour of analysis. Under these conditions 1 out of 21 cells died (also detailed in Table 2). (TIF) [file pone.0097565.s002.tif]

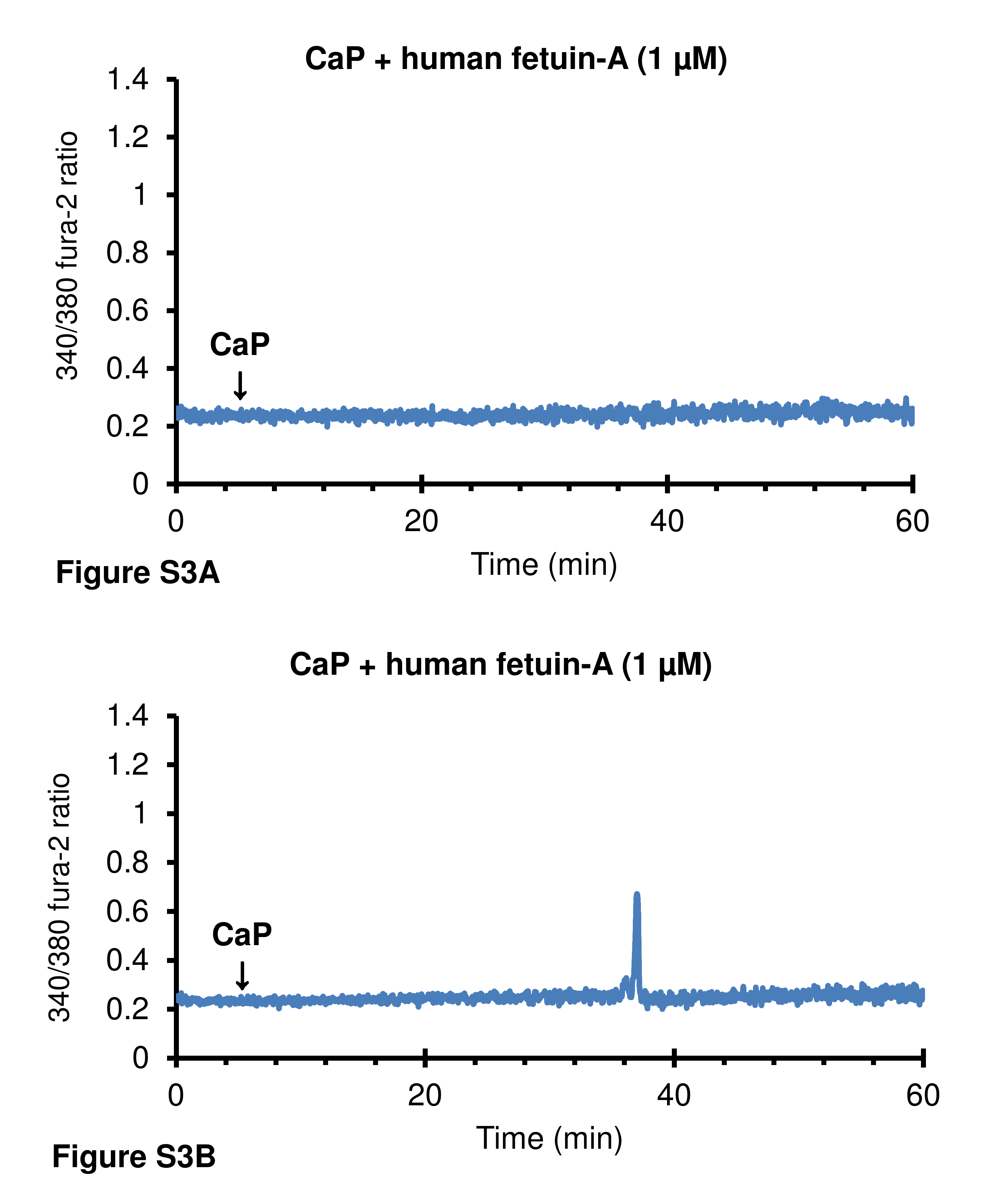

Supplement: Figure S3 — Effects of CaP particles on intracellular Ca2+ in the presence of human fetuin-A (1 µM). A and B are representative traces showing intracellular Ca2+ changes in individual fura-2-loaded VSMCs on addition of 25 µg/mL CaP particles (arrow indicates time of addition) in the presence of human fetuin-A (1 µM). Cells displayed either no intracellular Ca2+ changes (A) or clear intracellular Ca2+ spikes (B) over 1 hour of analysis. Under these conditions no cells died. (TIF) [file pone.0097565.s003.tif]

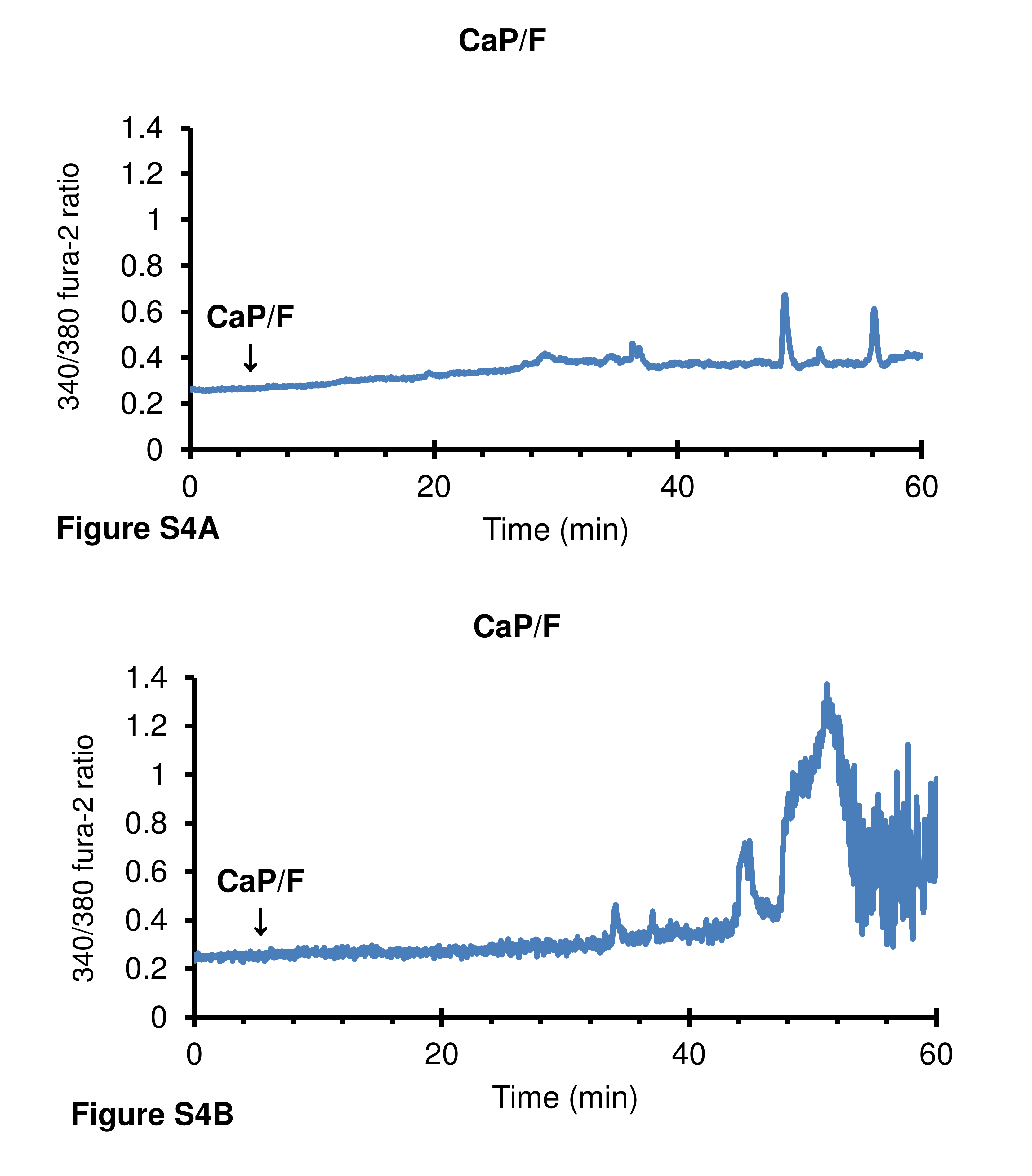

Supplement: Figure S4 — Effects of functionalised CaP/F particles on intracellular Ca2+. A and B are representative traces showing intracellular Ca2+ changes in individual fura-2-loaded VSMCs on addition of 25 µg/mL CaP/F particles (arrow indicates time of addition). All cells displayed intracellular Ca2+ changes in response to CaP/F particles over 1 hour of analysis. These intracellular Ca2+ responses were either non-toxic (40 out of 49 cells, 82% as in A) or toxic (9 out of 49 cells, as in B, also detailed in Table 2). (TIF) [file pone.0097565.s004.tif]

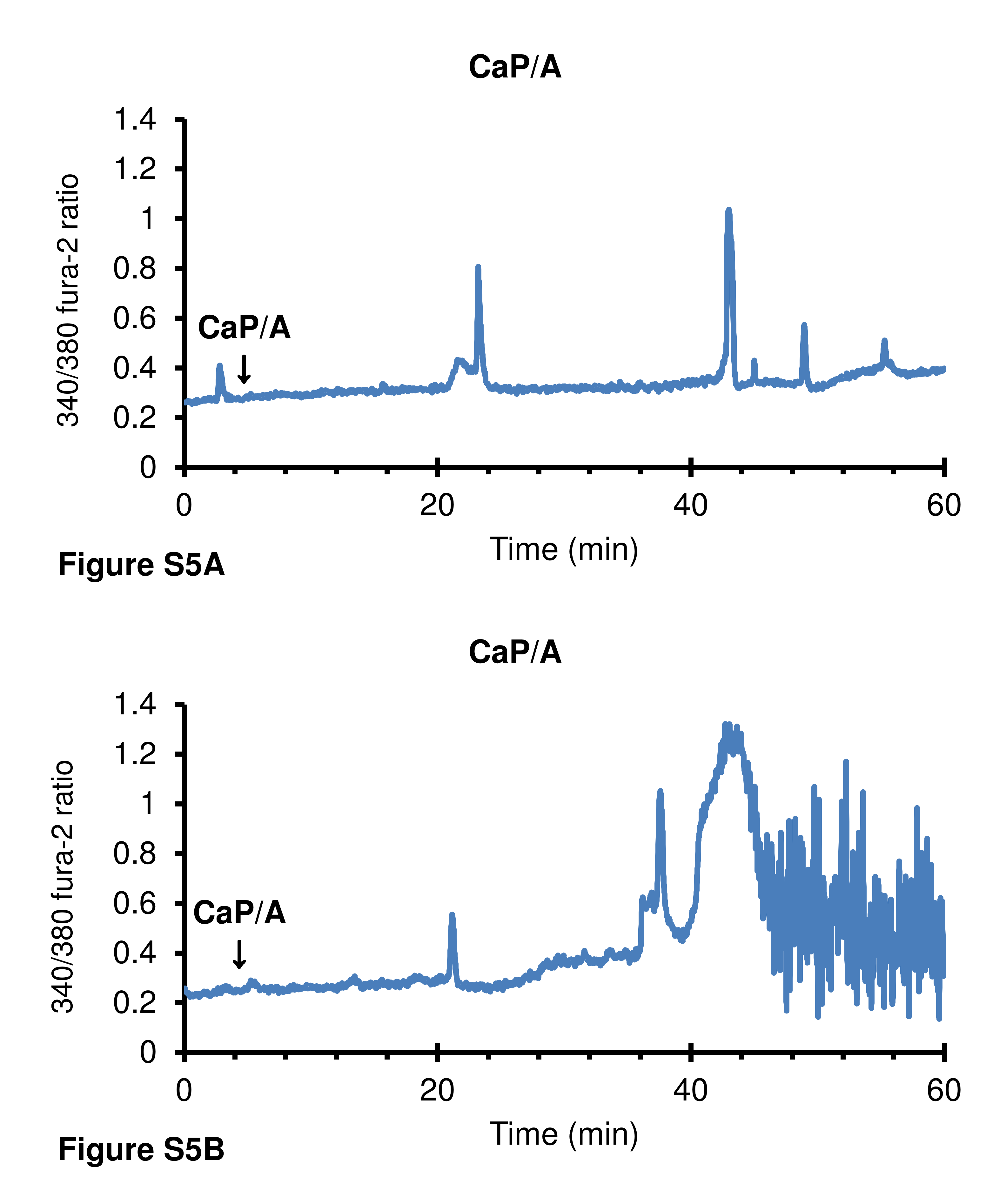

Supplement: Figure S5 — Effects of functionalised CaP/A particles on intracellular Ca2+. A and B are representative traces showing intracellular Ca2+ changes in individual fura-2-loaded VSMCs on addition of 25 µg/mL CaP/A particles (arrow indicates time of addition). All cells displayed intracellular Ca2+ changes in response to CaP/A particles over 1 hour of analysis. These intracellular Ca2+ responses were either non-toxic (2 out of 16 cells, 12% as in A) or toxic (14 out of 16 cells, 88%, as in B, also detailed in Table 2). (TIF) [file pone.0097565.s005.tif]

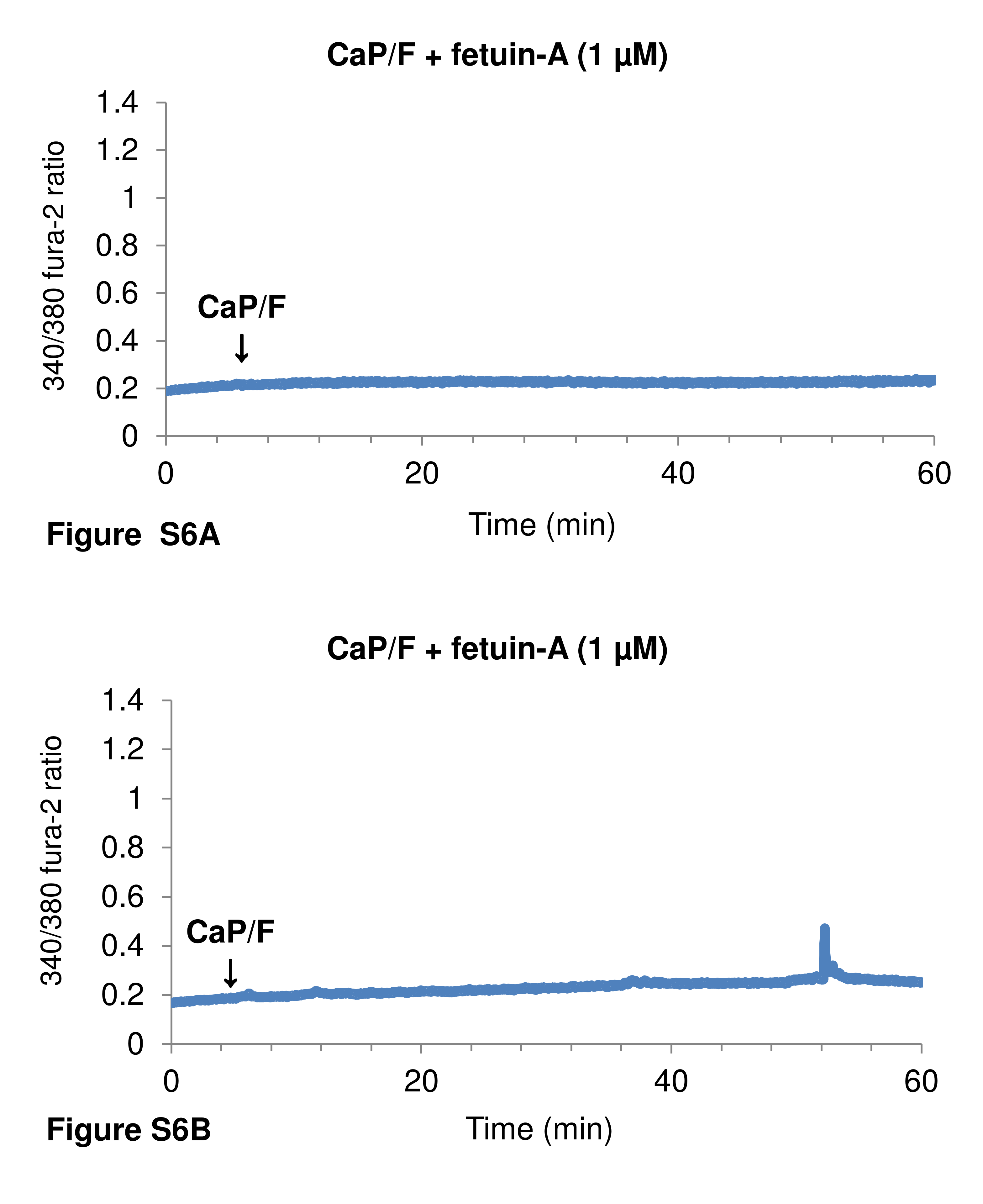

Supplement: Figure S6 — Effects of functionalised CaP/F particles on intracellular Ca2+ in the presence of fetuin-A (1 µM). A and B are representative traces showing intracellular Ca2+ changes in individual fura-2-loaded VSMCs on addition of 25 µg/mL CaP/F particles (arrow indicates time of addition) in the presence of fetuin-A (1 µM). No cells died with this treatment over 1 hour of analysis. Two different types of intracellular Ca2+ responses were observed, either no response (A), or cells displaying small changes in intracellular Ca2+ (B). (TIF) [file pone.0097565.s006.tif]
